# Mice have a transcribed L-threonine aldolase/GLY1 gene, but the human GLY1 gene is a non-processed pseudogene

**DOI:** 10.1186/1471-2164-6-32

**Published:** 2005-03-09

**Authors:** Alasdair J Edgar

**Affiliations:** 1 Department of Craniofacial Development, King's College, London, UK

## Abstract

**Background:**

There are three pathways of L-threonine catabolism. The enzyme L-threonine aldolase (TA) has been shown to catalyse the conversion of L-threonine to yield glycine and acetaldehyde in bacteria, fungi and plants. Low levels of TA enzymatic activity have been found in vertebrates. It has been suggested that any detectable activity is due to serine hydroxymethyltransferase and that mammals lack a genuine threonine aldolase.

**Results:**

The 7-exon murine L-threonine aldolase gene (GLY1) is located on chromosome 11, spanning 5.6 kb. The cDNA encodes a 400-residue protein. The protein has 81% similarity with the bacterium *Thermotoga maritima *TA. Almost all known functional residues are conserved between the two proteins including Lys242 that forms a Schiff-base with the cofactor, pyridoxal-5'-phosphate. The human TA gene is located at 17q25. It contains two single nucleotide deletions, in exons 4 and 7, which cause frame-shifts and a premature in-frame stop codon towards the carboxy-terminal. Expression of human TA mRNA was undetectable by RT-PCR. In mice, TA mRNA was found at low levels in a range of adult tissues, being highest in prostate, heart and liver. In contrast, serine/threonine dehydratase, another enzyme that catabolises L-threonine, is expressed very highly only in the liver. Serine dehydratase-like 1, also was most abundant in the liver. In whole mouse embryos TA mRNA expression was low prior to E-15 increasing more than four-fold by E-17.

**Conclusion:**

Mice, the western-clawed frog and the zebrafish have transcribed threonine aldolase/GLY1 genes, but the human homolog is a non-transcribed pseudogene. Serine dehydratase-like 1 is a putative L-threonine catabolising enzyme.

## Background

Elucidating the factors involved in threonine homoeostasis is important for the development of nutritional strategies in human clinical diets for treating patients suffering from wasting diseases. In farmed animals the regulation of livestock feed is required to ensure optimal growth and to reduce nitrogen excretion which poses environmental disposal problems. Threonine is required for protein synthesis and the removal of excess threonine by oxidation is needed to prevent its accumulation both intracellularly and in the circulation. The rate of catabolism of many amino acids, including threonine, increases when dietary protein exceeds the body's requirements. Gluconeogenesis occurs mainly in the liver where it helps maintain blood glucose homeostasis in mammals. During starvation amino acid catabolism increases to support gluconeogenesis. Glucocorticoids and glucagon hormones are known to up regulate and insulin down regulate the gene expression of many amino acid-catabolising enzymes [1].

There are three L-threonine (L-alpha-amino-beta-hydroxybutyric acid) degradation pathways in living organisms; via L-threonine aldolase (L-TA)(EC 4.1.2.5)(gene abbreviation GLY1), via L-serine/threonine dehydratase (SDH)(EC 4.2.1.16)(gene abbreviation SDS)(in bacteria also called L-threonine deaminase) and via L-threonine 3-dehydrogenase (EC 1.1.1.103)(TDH) [2-5]. L-threonine is broken down by; L-TA to yield glycine and acetaldehyde, by SDH to yield NH_4_^+ ^and 2-ketobutyrate and TDH to yield 2-amino-3-ketobutyrate. The subsequent reaction between 2-amino-3-ketobutyrate and coenzyme A to form glycine and acetyl-CoA is catalysed by 2-amino-3-ketobutyrate coenzyme A ligase (KBL)(EC 2.3.1.29), also called glycine acetyltransferase (gene abbreviation GCAT).

Together with the cofactor, pyridoxal-5'-phosphate (PLP), SDH uses threonine and serine as substrates to generate glycine which is used in gluconeogenesis. Serine dehydratase-like 1 gene (SDH1) is a second SDH gene found in vertebrates, but has yet to be characterised. I suggest that it is also a putative L-threonine catabolising enzyme.

Vitamin B_6_-dependant enzymes can be grouped according to their fold type. L-TA belongs to fold type I. L-TA enzymes are unrelated to D-TA enzymes which possess type III folds [6]. In vertebrates, the TA enzyme has not been purified by protein fractionation, only assayed in homogenised tissue fractions and isolated hepatocytes. In vertebrates most L-threonine degradation occurs via the enzymatic activities of serine/threonine dehydratase and threonine dehydrogenase. However, the presence of threonine aldolase enzymatic activity has been demonstrate in rat liver extracts [7-14]. Threonine aldolase contributes 1–3% of total threonine degradation under a variety of nutritional states in both rat and quail [4,15].

L-TAs from a number of species of bacteria and fungi have been isolated and characterized (reviewed in [16]). In the yeast, *Saccharomyces cerevisiae*, the glycine synthase-1 gene, GLY1 was identified as threonine aldolase [17,18]. Previously, gene ablation studies had shown that the GLY1 pathway is a major source of glycine [19]. But it only plays a minor role in *Candida albicans *[20]. In a number of bacteria species such as *Escherichia coli*, *Aeromonas jandaei*, *Pseudomonas *and *Thermatoga maritima *the GLY1 gene has been cloned and their enzymatic activity characterised [21-24]. In thale cress, *Arabidopsis thaliana*, there are two threonine aldolase genes (*THA1 *and *THA2*). *THA1 *has been shown to play a role in seed nutritional quality [25]. Putative GLY1 genes have been also identified in nematodes and flies [21]. Recently, the X-ray crystal structures of L-threonine aldolase from the bacteria *Thermotoga maritima *have been determined as the apo-enzyme, bound to L-*allo*-threonine and to glycine [21].

These GLY1/threonine aldolases are distinct from the serine hydroxymethyltransferases (EC 2.1.2.1)(SHMT). However, some SHMT also possess some threonine aldolase enzymatic activity. SHMT from *E. coli *and the yogurt bacterium, *Streptococcus thermophilus*, have TA activity [26,27]. SHMT isolated from rabbit liver has been shown to possess weak TA activity [28]. Consequently, it has been thought that the minor threonine aldolase activity in liver extracts was due solely to SHMT, and that mammals lack a true threonine aldolase, but this has been questioned [29]. Here I report that TA genes are present in vertebrates.

## Results

### Analysis of murine L-threonine aldolase cdnas

**Figure 1:**
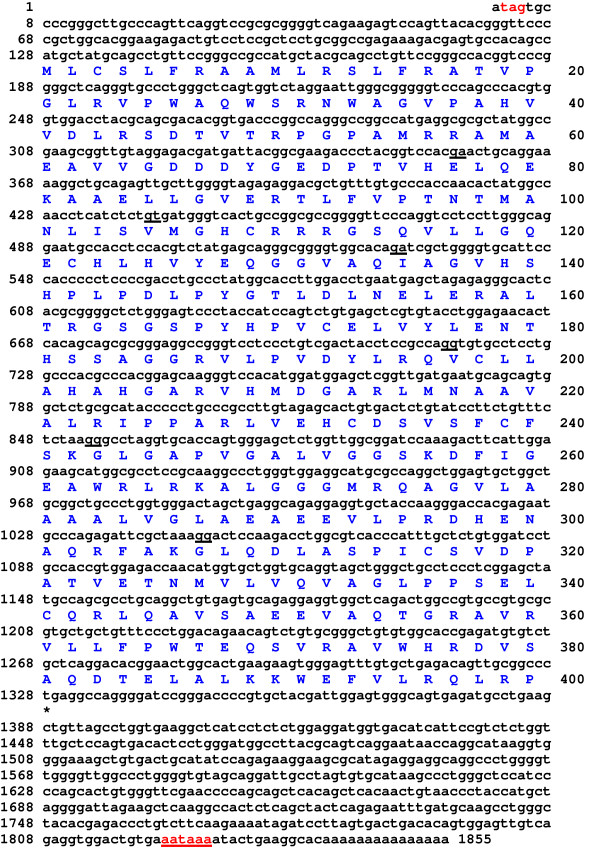
The cDNA sequence and translation of murine L-threonine aldolase. A potential polyadenylation signals (aataaa at 1822–1827) is shown in bold and underlined with the polyadenylation sites indicated by a. An * indicates the tga stop codon. The underlined nucleotide pairs indicate the positions of the exon/exon boundaries. The in-frame stop codon in the 5'UTR is indicated, **tag**, (coloured red).

**Figure 2:**
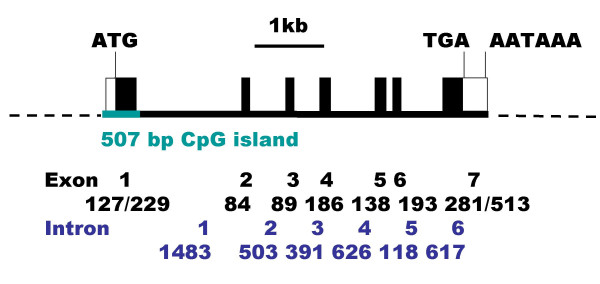
Chromosomal localisation and the gene structure of murine L-threonine aldolase gene. (A) The gene is located on chromosome 11, band E2 (accession No. AL591433, the Sanger Institute, UK). (B) The 7-exon gene spans 5.6 kb. There is a CpG island spanning the 5' untranslated exon. The ORF is indicated by closed boxes. The sizes, in bp, of the exons and introns are indicated.

I conducted a search of the GenBank database for a putative mouse L-threonine aldolase gene using the sequence of the *E. coli *TA protein [22]. PCR primers were designed to the 5' and 3' ends of EST sequences that matched the genomic DNA sequence of the putative L-threonine aldolase gene. These primers were used to amplify the cDNA from murine liver RNA by RT-PCR. The amplicons were electrophoresised on an agarose gel. Two bands of similar intensity were obtained. Both bands were excised from the gel, cloned and sequenced. The upper band encoded an 1855 bp murine L-threonine aldolase cDNA sequence. It has a 127 bp 5'UTR containing an in-frame stop codon, an ORF which encodes a 400 residue protein and has an ATTAAA polyadenylation signal at 1822–1827 (GenBank accession No. AY219871)(Fig. 1). The predicted protein has a 43,496 Da molecular mass and an isoelectric point 6.73. The lower band encoded a second cDNA clone that was identical to the first clone except that it skipped exon 3. On translation, this results in a frame shift in the ORF that would encode a severely truncated protein of 124 residues that would not be expected to have any enzymatic activity (GenBank accession No. AY219872). Both cDNA sequences matched the mouse genomic DNA sequence. The mouse L-threonine aldolase/Gly1 gene is located on chromosome 11 band E2 (clone RP23-268N22, EMBL accession No. AL591433, Sanger Institute, UK) towards the telomere, between the baculoviral IAP repeat-containing 5 (Birc5) and suppressor of cytokine signalling 3 (Socs3) genes. The L-threonine aldolase gene spans 5.6 kb, consisting of 7 exons (Fig. 2). All splice donor/acceptor sites have consensus GT/AG dinucleotides. There is a 507 bp CpG island (66% GC) encompassing exon 1. Such CpG islands are generally associated with active housekeeping genes [30]. The predicted start of transcription, CCAT, on the genomic DNA is just 2 bp upstream of the cDNA sequence suggesting that the clone is almost full-length.

### Predicted secondary structure of the murine threonine aldolase protein

**Figure 3:**
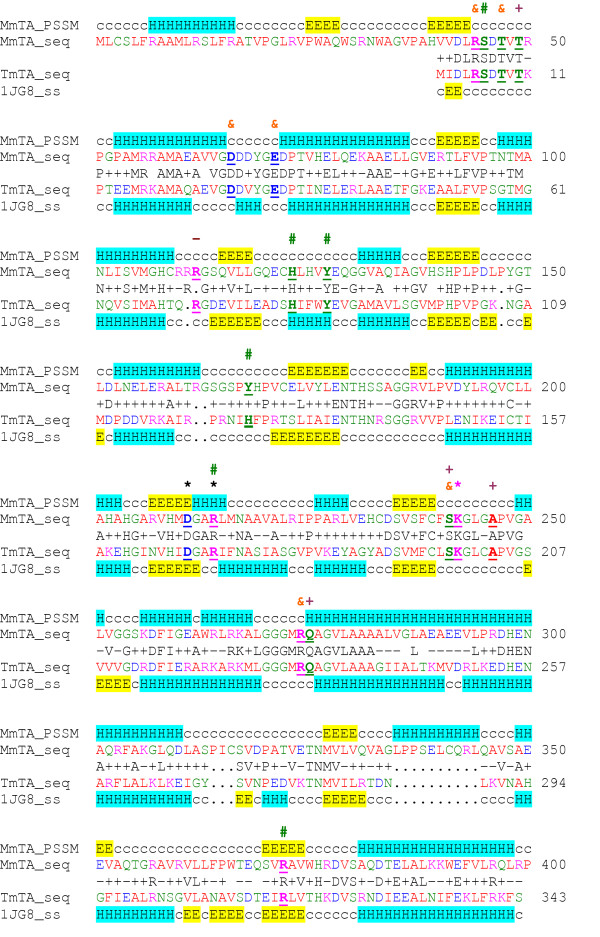
Comparison of the predicted secondary structure of the murine threonine aldolase protein with that of the crystal structure of threonine aldolase from the bacteria *Thermotoga maritima*. The labels are: mouse predicted secondary structure, MmTA_PSSM; mouse protein sequence, MmTA_seq; *T. maritima *protein sequence, TmTA_seq and *T. maritima *secondary structure 1JG8_ss; alpha-helix, H, highlighted in light blue; beta-sheet, E, highlighted in yellow; c = turn, coil or loop. Identical residues in both proteins are illustrated with a "+" indicating positive equivalence and a "-" a negative equivalence. The PLP-binding lysyl residues are indicated with a pink asterisk and those residues that interact with PLP are indicated with a black asterisk. Those residues that contact the substrates, L-threonine and L-*allo*-threonine, and the product, glycine, are indicated with a green hash. Residues involved in electrostatic interactions in the catalytic dimer interface are indicated with an ampersand. Residues making contact with calcium ions are indicated with a plus sign and those contacting a chloride ion with a negative sign.

A comparison of the predicted secondary structure of the murine TA protein with the known secondary structure of *T. maritima *[21] is shown (Fig. 3). The proteins have 44% identity and 81% similarity and are similar throughout their length. Overall there is good correspondence between the position of the predicted α-helices and β-sheets in the murine protein with those determined from the crystal structure of *T. maritima*. However, the mouse protein has an additional putative amino-terminal mitochondrial import leader peptide. Given long evolutionary distance between mouse and bacteria this high degree of homology strongly suggests that this murine protein is also a threonine aldolase. Most functional residues are conserved between the two proteins. By homology with the *T. maritima *protein, Lys242 is expected to form a Schiff-base with the cofactor, pyridoxal-5'-phosphate (PLP), with Asp211 and Arg214 expected to interact with PLP. Those residues that contact the ligands L-*allo*-threonine and glycine, Ser45, His123, Tyr127, Arg214 and Arg372, are conserved. *T. maritima *His125 from the second subunit is predicted to bind the hydroxyl group of L-threonine. This residue is homologous to murine Tyr168, a conservative substitution since both residues are polar and aromatic. In other TA proteins from diverse phyla this residue is mainly histidine, but in rice it is a tyrosine also. At the catalytic dimer interface electrostatic interactions occur among the side chains of Arg44-Glu71, Thr47-Asp66 and Arg274-Ser241. These residues are conserved. Residues involved in ion coordination are also conserved with Ala246, Thr49 and Ser241 contacting Ca^2+ ^with Arg112 contacting a chloride ion. In the Arabidopsis *THA1 *enzyme a Gly114 to Arg mutation, located between two beta-sheets, results in loss of enzymatic activity [25]. This residue, Gly149 in mouse, is conserved in all four vertebrate TA enzymes.

### Sequence homology to other vertebrate threonine aldolase proteins

**Figure 4:**
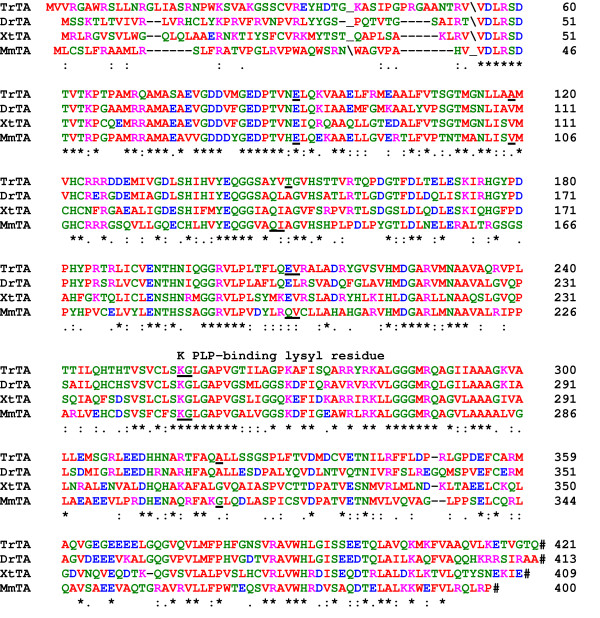
Comparison of vertebrate L-threonine aldolase protein sequences. The L-threonine aldolase sequences are: mouse, *Mus musculus*, MmTA; pufferfish, *Takifugu rubripes*, TrTA; (BK005561); western clawed frog, *Xenopus tropicalis*, XtTA (BK005562) and zebrafish, *Danio rerio*, DrTA (AAH72718). Predicted cleavage sites during import into mitochondria are indicated by a backwards slash (\). Where known, the locations of the exon/exon boundaries are shown on the translated protein as underlined residues. Stop codons are indicated by a hash. Conserved residues are indicated by a (*), strongly similar residues by a (:) and weakly similar residues by a (.). Residues are colour coded: basic, DE, red; acidic, KR, pink; polar, CGHNQSTY, green and hydrophobic, AFILMPVW, red.

Database searches revealed the presence of other L-threonine aldolase genes in other vertebrates (Fig. 4). There is a single seven exon gene in the Japanese puffer fish genome (*Takifugu rubripes*)(accession No. BK005561). The exon/exon boundaries on the proteins are highly conserved between mouse and Japanese puffer fish with only one being displaced slightly. The Japanese puffer fish gene encodes a 421-residue protein that has 46% identity and 74% similarity to the murine protein. Similar L-threonine aldolase cDNAs for the western-clawed frog and the zebrafish were identified (accession Nos. BK005562 and AAH72718 respectively). The proteins are of similar lengths with the functional residues identified in *T. maritima *being well conserved. Homology extends throughout their lengths, apart from the amino-terminal regions. Despite their low sequence identity in the amino-terminal region all four proteins contain putative mitochondrial import leader peptides, being positively charged. They possess also a predicted cleavage site that would be utilised during their import into mitochondria. After cleavage of the mitochondrial import sequence the mature murine TA enzyme would have a mass 39,778 Da and pI 6.11. Recently, two other vertebrate GLY1 genes have been sequenced. In the dog there is a complete gene (GenBank accession number NW_140385) that would encode a 391-residue protein with 82% identity and 94% similarity to murine TA. Three overlapping unassigned genomic DNA sequences from the freshwater puffer fish, *Tetraodon nigroviridis*, would encode a gene encoding a 431-residue protein with 44% identity and 73% similarity to murine TA. Additionally, homologous coding ESTs from mammals (rat, pig and cow), birds (chicken), amphibians (African clawed frog) and fish (little skate, rainbow trout, Atlantic salmon, channel catfish and Japanese medaka) were identified, indicating that L-threonine aldolase expression in vertebrates is widespread.

### Human GLY1/threonine aldolase gene is a pseudogene

A database search identified 17q25 as the location for the human GLY1 gene. All exon/intron boundaries found in the murine threonine aldolase gene are conserved in man. The mouse to human conserved synteny map shows that both GLY1 genes have the synaptogyrin 2 (SYNGR2), baculoviral IAP repeat-containing 5 (BIRC5) and soluble thymidine kinase 1 (TK1) genes as near neighbours. Starting with human liver RNA, I was neither able to amplify any threonine aldolase transcripts using a variety of 5' and 3' RACE methods nor to detect any transcripts in a wide range of tissue and cell line cDNAs by RT-PCR. A search of the human EST database identified five potential EST transcripts scattered throughout the threonine aldolase gene, but they lack supporting evidence that they are truly transcribed sequences, being unspliced singletons. There is a potential polyadenylation signal site in the human threonine aldolase gene with good homology to the murine site. However, corresponding 3'UTR ESTs and SAGE tags are conspicuously absent from the databases leading to the conclusion that the GLY1 threonine aldolase gene is not transcribed in man.

**Figure 5:**
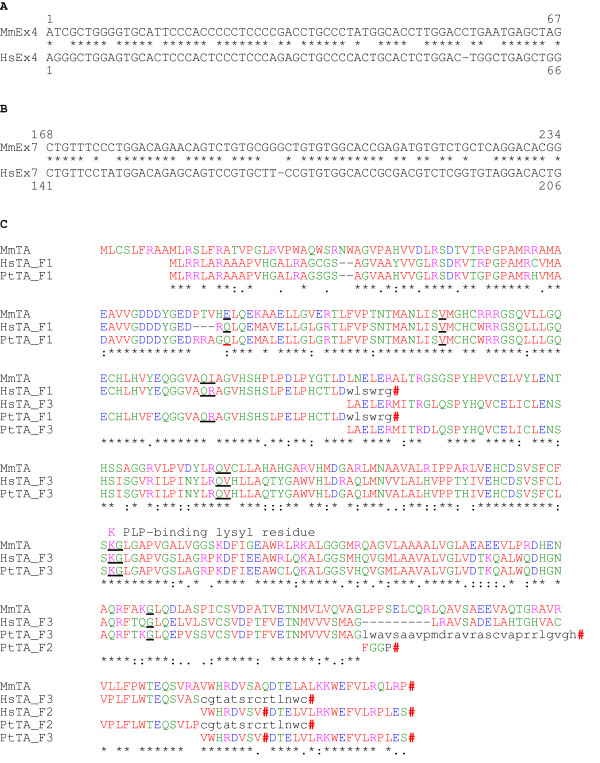
Comparison of the mouse threonine aldolase cDNA and ORF with the human and chimpanzee genes. (A) There is a cytidine deletion in exon 4 of the human threonine aldolase gene resulting in a frame-shift. (B) There is a guanosine deletion in exon 7 of the human threonine aldolase gene resulting in a frame-shift. (C) Comparison of the mouse protein with a translation of human and chimpanzee genes shows that the presence of the frame-shift in exon 4 creates a truncated ORF of 144 residues that does not include the PLP-binding lysine residue (pink K); consequently the protein would be non-functional. All exon/exon boundaries are conserved and shown on the translated protein as black underlined residues except that of chimpanzee exon 1 which is shown as a red underlined residue. Stop codons are indicated by red hashes. ORF residues generated by frame-shifts are shown in lower case. Conserved residues are indicated by a (*), strongly similar residues by a (:) and weakly similar residues by a (.). Abbreviations: mouse, *Mus musculus*, Mm; *Homo sapiens*, Hs; Pt, *Pan troglodytes*; exon 4, Ex4; exon 7, Ex7; translations in frame 1, F1; frame 2, F2 and frame 3, F3.

If the human GLY1 threonine aldolase gene were transcribed there are two single nucleotide deletions that would cause frame-shifts. They are the equivalent of murine nucleotide 55 in exon 4 (Fig. 5A) and the equivalent of murine nucleotide 198 in exon 7 (Fig. 5B). Both these deletions are found in genomic DNA clones from two individuals showing that these deletions are not sequencing errors (accession Nos. AC032035 and AC010532, MIT Center for Genome Research, USA and DOE Joint Genome Institute, USA, respectively). The presence of the frame-shift in exon 4 would create a truncated ORF of 144 residues that does not include the PLP-binding lysine residue, consequently the protein would not be functional (Fig. 5C). Also there is a premature in-frame stop codon towards the carboxy-terminal. Even if the frame-shifts in the human GLY1 gene were not present then the translated human TA protein would not function due to the mutation of four important residues. These four residues have remained conserved during evolution since the last common ancestor of the bacteria, *T. maritima*, and vertebrates. One residue that would be expected to interact with the PLP ligand, murine Arg214, would be mutated to Gln in man. Murine residue Arg372 that would be expected to interact with threonine is mutated to Ala. The side chains of two residues that form electrostatic interactions at the catalytic dimer interface are also mutated, murine Thr47 to Lys, and murine Arg274 to His. If the frame-shifts were not present, the mouse and human proteins would have 66% identity and 85% similarity. Likewise, the chimpanzee threonine aldolase gene is a pseudogene possessing the same frame-shifts as the human gene. Additionally, it has lost the splice donor site in exon 1 and, by comparison with the mouse gene, has a 64 bp deletion in exon 7 (Fig. 5C).

### Homology of serine/threonine dehydratase and serine dehydratase like-1 proteins in vertebrates

**Figure 6:**
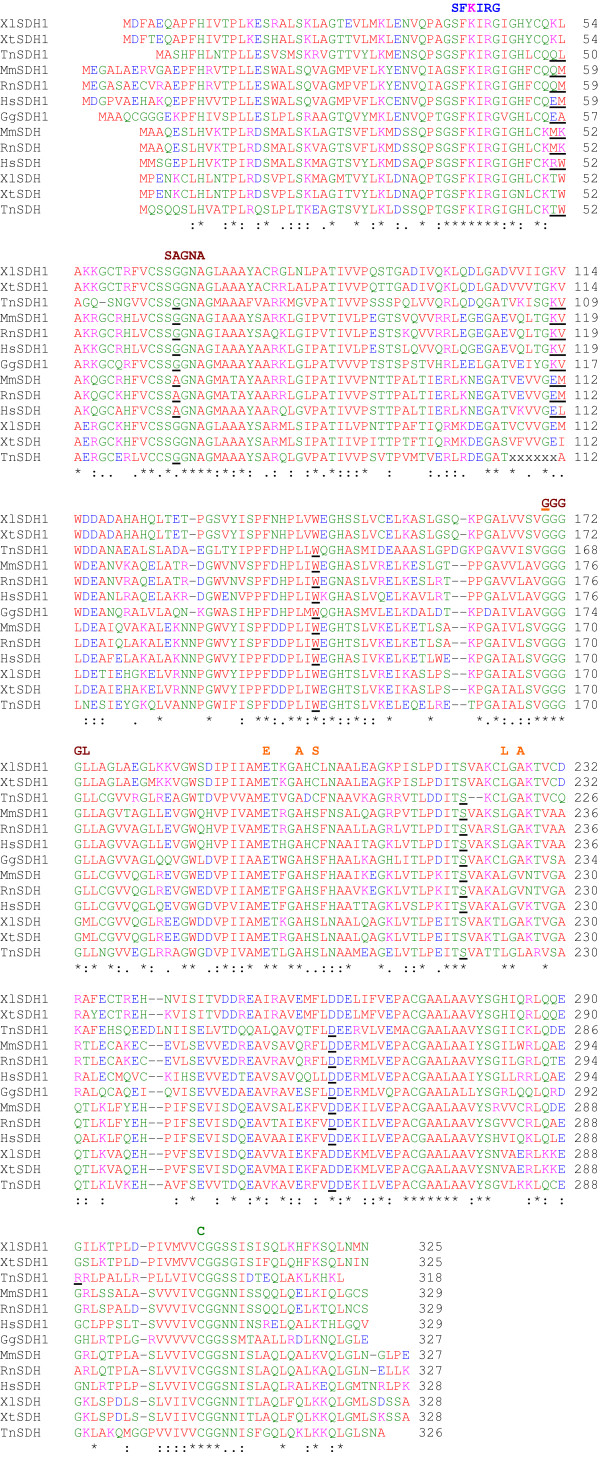
Comparison of vertebrate SDH and SDH1 proteins. The species are: house mouse, *Mus musculus*, Mm; Norway rat, *Rattus norvegicus*, Rn; human, *Homo sapiens*, Hs; chicken, *Gallus gallus*, Gg; western clawed frog, *Xenopus tropicalis*, Xt; African clawed frog, *Xenopus laevis*, Xl; freshwater puffer fish and *Tetraodon nigroviridis*, Tn. By comparison with the crystal structure of rat SDH, important conserved residues found in SDH enzymes are conserved also in SDH1 and are shown above the sequence alignment. The amino acid sequence SFKIRG (blue), around the PLP binding Lys41 (pink), is conserved in SDH and SDH1. Two other conserved amino acid sequences, SAGNA (brown) and GGGGL (purple), form hydrogen bonds with PLP, as does Cys303 (green). A potassium ion near the active site is coordinated by six oxygen atoms from Gly168, Ala198, Leu223, Val225, Glu194, and Ser200 (orange). Conserved residues are indicated by a (*), strongly similar residues by a (:) and weakly similar residues by a (.). Residues are colour coded: basic, DE, red; acidic, KR, pink; polar, CGHNQSTY, green and hydrophobic, AFILMPVW, red. Exon/exon boundaries determined from genomic DNA are indicated on the proteins by black underlining. Unknown sequences are indicated by xx.

The sequences of murine SDH and SDH-1 cloned cDNAs matched those of reference sequences (accession numbers NM_145565 and NM_133902 respectively). In mammals, these two genes are adjacent, being arranged in a 5' to 5' orientation. Database searches identified both the SDH and SDH1 genes in man, rat, freshwater puffer fish and the Western and African clawed frogs. But in the chicken only the SDH1 gene is present since SDH is absent from the draft genome and all expressed sequences. A comparison of vertebrate SDH and SDH1 proteins with the crystal structure of rat SDH [31] suggests that SDH1 is also a serine/threonine dehydratase because residues with important functions are conserved (Fig. 6). By homology, Lys48 of murine SDH1 is the PLP binding residue forming a Schiff base and the amino acid sequence around Lys48 SxKIRG is well-conserved in other SDHs from vertebrates, plants, yeasts and bacteria [32-35]. Two other conserved amino acid sequences, S(A/G)GNA and GGGG(L/M) and Cys309 (murine SDH1 numbering) form hydrogen bonds with PLP. In SDH1 a potassium ion near the active site would be expected to be coordinated by six oxygen atoms, five of which are from conserved residues; Gly174, Glu200, Ala204, Ser206, Leu229, but Ala231 replaces Val225 of rat SDH.

### Expression of threonine aldolase, serine/threonine dehydratase and serine dehydratase like-1 mRNA in mouse tissues

**Figure 7:**
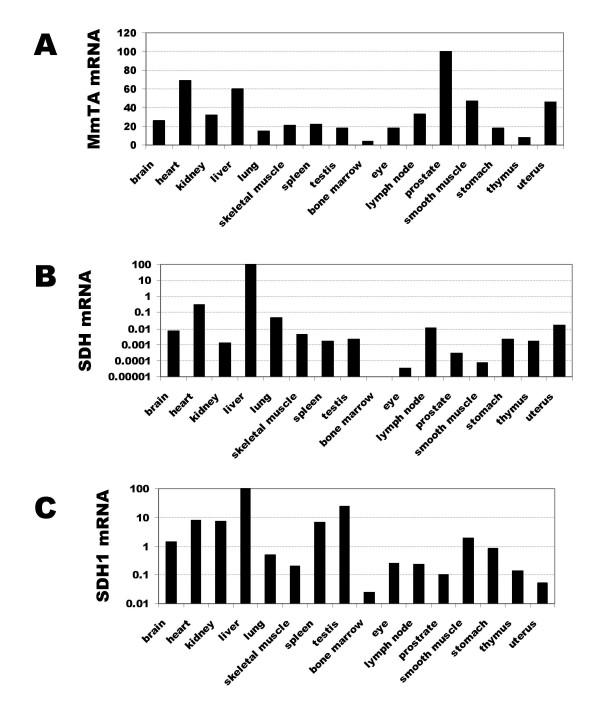
Expression of TA, SDH and SDH1 mRNA in mouse tissues by real time PCR. The expression levels in each tissue were normalised to that of the housekeeping genes beta-actin and G3PDH. (A) For TA the expression levels in all tissues were standardised to that of prostate, which was taken as 100; (B) and for SDH and SDH1 the expression levels were standardised to that of liver, which was taken as 100.

To identify those tissues which are likely to contribute to TA activity in the mouse, the expression of TA mRNA in adult tissues was examined by RT-real time PCR normalised to the expression of the housekeeping genes, β-actin and glyceraldehyde-3-phosphate dehydrogenase (G3PDH). Low levels of TA mRNA were detected in all tissues examined. They varied 20-fold between tissues, being highest in prostate, heart and liver (Fig. 7A). In contrast, the mRNA levels of SDH, another enzyme that catabolises L-threonine, has a very specific tissue distribution. It is expressed highly in the liver at a level similar to the two housekeeping genes. It is over 300 fold more abundant in liver than heart, the second highest expressing tissue (Fig. 7B). Low levels of SDH1 mRNA were found also in all tissues. Like SDH, SDH1 was most abundant in the liver with moderate levels being found in testis, heart, kidney and spleen (Fig. 7C).

### Expression of threonine catabolic enzymes in mouse embryos

**Figure 8:**
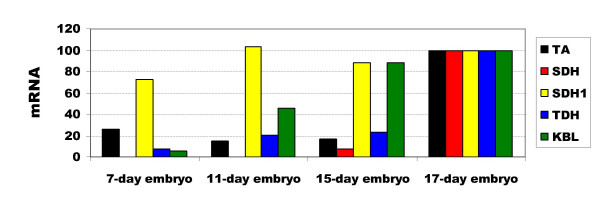
Expression of TA, SDH, SDH1, TDH and KBL mRNA in whole mouse embryos by real time PCR. Their expression levels in each embryonic stage were normalised to that of the housekeeping genes beta-actin and G3PDH. Each gene was standardised to its expression level at embryo day 17, which was taken as 100. The gene abbreviations are: threonine aldolase, TA; serine/threonine dehydratase, SDH; serine dehydratase like-1, SDH1; threonine dehydrogenase, TDH and 2-amino-3-ketobutyrate coenzyme A ligase, KBL.

The mRNA expression of threonine catabolic enzymes was examined by real time PCR in cDNAs derived from whole mouse embryos from days 7, 11, 15 and 17 (Fig. 8). Overall, TA, TDH and SDH expression were low prior to E-15, but increased more then four-fold by E-17. KBL expression was low at E-7, but increased earlier than the other enzymes. SDH1 did not change substantially with increasing embryonic age.

## Discussion

In vertebrates, L-threonine is one of the indispensable amino acids. It is obtained from protein in the diet, typically being the second or third limiting amino acid in herbivorous diets. Some of it is utilized in synthesising new protein, but the rest is converted to other amino acids by oxidative catabolism by three different enzymes that are found in most organisms; TDH, TA and SDH. Both the TDH and TA pathways produce glycine. However, the TDH pathway occurs in two steps, requiring KBL as the second step. Using protein homology searches of the mouse genome with the bacterial enzymes has allowed me to identify and clone TDH and KBL cDNAs [36,37]. GLY1/TA genes have been identified previously in bacteria, fungi and plants [16,21,25]. Here I describe the first TA cDNA found in vertebrates. The murine TA cDNA encodes a 400-residue protein that is highly similar to that from *T. maritima *with an expect value of 2e^-73^, being clearly distinct from glycine dehydrogenase, the second most closely related protein, with an expect value of 0.002. This remarkable conservation, over billions of years of evolution since the last common ancestor, shows the general importance of these metabolic pathways. However, the presence of some abnormal TA mRNA splicing in mouse, the low levels of mRNA found in mouse tissues, together with the low levels of TA enzymatic activity found in rat liver [4,15] plus the loss of a functional TA gene in humans suggests that TA has reduced importance in mammals.

The L-TA enzymes can act on the stereoisomers, L-threonine and L-*allo*-threonine. These can be divided into three types based on the stereospecificity towards the β-carbon of threonine. Low-specificity L-TA can use both L-threonine and L-*allo*-threonine as substrates. L-TA only acts on L-threonine and L-*allo*-TA is specific to L-*allo*-threonine [16]. Murine L-TA is likely to be a low-specificity L-TA with a preferences for the *allo *isomer in a manner similar to the *T. maritima *enzyme, because Tyr127 (Tyr87 in *T. maritima*) in the TA active site is conserved, a residue which appears to be involved in discriminating L-threonine from L-*allo*-threonine [21].

All the vertebrate TA proteins have putative amino-terminal mitochondrial import sequences, suggesting that the mitochondrion is its intracellular localisation. In contrast, fractionation studies in the yeast, *S. cerevisiae*, revealed a cytosolic localisation for TA [38]. Additionally, the yeast TA protein does not possess an amino-terminal mitochondrial import sequence. In vertebrates, threonine catabolism is mostly confined to the liver when the mass of the organ is taken into consideration. Expression of SDH, TDH and KBL mRNA are highest in liver [36,37,39]. However, low levels of murine TA expression were found in a wide range of tissues suggesting a role in housekeeping metabolism in all tissues. Generally, during embryogenesis, expression of threonine catabolic enzymes increased with maturation of the developing liver.

Humans have lost two of the three enzymes of threonine catabolism with both GLY1 and TDH [40] genes being defective, both pathways produce glycine from threonine. In man, the loss of a functional GLY1 gene appears to be a more ancient event than the loss of TDH because GLY1 genes in both man and chimpanzees have a number of frame-shifts and mutations of functional amino acid residues, whereas the mutated exon 6 splice-acceptor site in human TDH is intact in chimpanzees (data not shown). This suggests that GLY1 has been lost prior to, and TDH after, the divergence of man and chimpanzees, about 6–8 million years ago. Consequently, humans may not be as metabolically well equipped as other species to cope with diets high in threonine/protein. Perhaps a reduction in the rate of threonine catabolism in man's ancestors would have conferred a selective advantage on those individuals with these defective genes under conditions of protein starvation. Although humans have lost both the glycinergic pathways of threonine catabolism their gut microbial flora will have both TA and TDH enzymes, therefore, gut microbial flora may make significant contributions to human threonine catabolism.

With the loss of TA and TDH genes in humans this leaves serine/threonine dehydratase as our only major threonine catabolic enzyme. However, vertebrates also have a second SDH gene, called SDH-like-1 which, by homology, is likely to function also as a serine/threonine dehydratase since all the residues that bind the PLP co-factor are conserved between the two proteins. Only the SDH1 gene is present in the chicken, therefore the serine dehydratase activity found in chick livers [41] must be due to SDH1.

The SHMT enzymes are members of the α-class of pyridoxal phosphate enzymes, catalyzing the reversible interconversion of serine and glycine. Mammals have two SHMT genes. One encodes a cytosolic and the other a mitochondrial enzyme. Purified SHMT enzyme from rat liver possesses some threonine aldolase activity [28] and both SHMT genes may also contribute to threonine catabolism in vertebrates.

With the identification of murine TA and SDH-1 mRNA the way is open to study their enzymatic activity *in vitro *and relative contribution to threonine catabolism under different physiological states *in vivo*. Changes in TA and SDH-1 mRNA expression in response to diet have yet to be examined, but rats fed a high protein diet or fasted showed an increase in TA enzymatic activity [42]. In contrast, quails and rats fasted or on threonine enriched diets did not show any statistically significant changes in TA enzymatic activity [15].

## Conclusion

I have shown that GLY1/TA genes are present in vertebrates. TA genes and enzymatic activities have been previously isolated from bacteria, fungi and plants. These enzymes are distinct from the serine hydroxymethyltransferases. The mouse GLY1 gene is located on chromosome 11, band E2 and the 1855 bp cDNA from this gene encodes a 400-residue threonine aldolase. The presence of a positively-charged amino-terminal import leader peptide sequence in mammalian, amphibian and fish TA proteins, that are not present in bacterial proteins, suggests that the vertebrate TA enzymes are mitochondrial. Man and chimpanzees have lost a functional GLY1 gene. Vertebrates also have a second SDH gene, SDH1, that by homology to the crystal structure of SDH may function as a threonine dehydratase and contribute to threonine catabolism.

## Methods

### Molecular cloning of murine L-threonine aldolase

Total RNA was extracted from mouse liver using guanidine thiocyanate and treated with DNase-I to remove any contaminating genomic DNA (SV total RNA isolation system, Promega, UK). Total RNA was reversed transcribed with AMV RNase H- reverse transcriptase (ThermoScript, Life Technologies, UK) at 50°C using an oligo-dT primer. The cDNA was amplified by touchdown PCR using the Advantage cDNA polymerise mix (Clontech, UK) on a Perkin-Elmer 2400 thermocycler. Amplification conditions for the first 10 cycles were 94°C for 5 sec, 72°C less 0.4°C per cycle for 3 min and for the next 20 cycles 94°C for 5 sec, 68°C for 10 sec, 72°C for 3 min per cycle using primers (100 nM) derived from the sequence of the mouse genomic DNA from clone RP23-268N22 (accession number AL591433 forward 5'-ATAGTGCCCCGGGCTTGC-3' and, first reverse 5'-TTTTTTTTTTTTTTTGTGCCTTCAGTATTT-3' and (Amersham-Pharmacia Biotech, UK). PCR amplicons were electrophoresised in a low-melting point agarose gel stained with ethidium bromide. They were excised from the gel. The agarose digested with agarase (Promega, U.K.). These PCR amplicons were cloned into pCR-II-TOPO, a T-A vector (Invitrogen, The Netherlands) and sequenced in both directions using the big dye terminator cycle sequencing ready reaction kit with AmpliTaq DNA polymerase FS on an ABI 373XL Stretch Sequencer (both from PE Applied Biosystems, UK). SDH and SDH-1 cDNAs were cloned in a similar manner and were used as positive controls in RT-PCR assays.

### Gene expression in mouse tissues by real time PCR

Quantitative PCR was carried out on a GeneAmp 5700 Sequence Detection System (AB Applied Biosystems) and a Rotor Gene 3000 utilising a CAS-1200 robotic precision liquid handling system (Corbett Research, Australia) using a SYBR Green I double-stranded DNA binding dye assay (Applied Biosystems, UK). For the determination of TA, SDH, SDH1, TDH and KBL mRNA expression in adult mouse tissues and whole mouse embryos, cDNAs were generated from polyA^+ ^selected RNA by reverse transcriptase using an oligo-dT primer (BD Clontech, UK). Approximately 8.0 ng of cDNA were used for each PCR. Tissue master mixes were divided into gene specific mixes and primers were added to a final concentration of 200 μM. The primers were: TA, CCCAGAGATTCGCTAAAGGACTC (exon 6/7) and CACGGCCAGTCTGAGCCAC (exon 7), which produced a 171 bp amplicon; SDH, TTTTACGAACACCCCATTTTCTC (exon 7) and AGAATCTTCTCATCGTCCACGAA (exon 8/7), which produced an 89 bp amplicon; SDH1, CCTGCCAGACATCACCAGTGT (exon 6/7) and GCGCTCATCGTCCAGGAA (exon 8/7), which produced a 154 bp amplicon; G3PDH, TCCCACTCTTCCACCTTCGA and GTCCACCACCCTGTTGCTGTA, which produced a 111 bp amplicon. Primers for beta-actin, TDH and KBL have been described previously [36]. Amplification conditions were; a 10 min hot start to activate the polymerase followed by up to 50 cycles of 95°C for 15 sec and 60°C for 1 min. The number of cycles required for the fluorescence to become significantly higher than background fluorescence (termed cycle threshold [C_t_]) was used as a measure of abundance. A comparative C_t _method was used to determine gene expression. Expression levels in each tissue cDNA sample were normalised to the average expression levels of the housekeeping genes beta-actin and G3PDH (ΔC_t_). Ratios of gene of interest mRNA/housekeeping mRNA from each tissue were standardised to that of the highest expressing tissue for that gene which was taken as 100% (ΔΔC_t_). Formula E^-ΔΔCt ^was used to calculate relative expression levels where E is the efficiency of the PCR per cycle. Amplification specificity was confirmed by melting curve analysis and agarose gel electrophoresis.

### Bioinformatics

The predicted start site of transcription of murine TA mRNA was determined using the program Eponine [43]. The predicted secondary structure of the protein was determined using the Psi-Pred program [44] aligned with that of the crystal structure of TA from the bacteria *T. maritima *[21] using 3D-PSSM [45]. Mitochondrial locations were predicted for the TA proteins using MITOPRED [46]. Cleavage-sites in the mitochondrial targeting peptides were identified using PSORT [47].

## Authors' contributions

A.J.E. initiated and carried out the molecular genetic studies, drafted the manuscript and approved the final manuscript.
